# Neglected tropical diseases: A proxy for equitable development and shared prosperity

**DOI:** 10.1371/journal.pntd.0005419

**Published:** 2017-04-20

**Authors:** Dirk Engels

**Affiliations:** Department of Control of Neglected Tropical Diseases, World Health Organization, Geneva, Switzerland; Baylor College of Medicine, Texas Children's Hospital, UNITED STATES

Neglected tropical diseases (NTDs) prevail among poor people in developing countries who that largely remain out of reach of national health care delivery systems. Since 2005, the WHO has moved away from a disease-centred to an integrated, intervention-based approach to counter these diseases, providing marginalised communities with better access to health care [1].

In 2007, a meeting of the WHO’s global partners marked a turning point in efforts to overcome NTDs, resulting in an increased commitment and engagement from endemic WHO Member States and the pharmaceutical industry and expanded collaboration among partners [2].

In 2012, inspired by the WHO Roadmap on NTDs, multiple stakeholders, including chief executive officers from the world’s major pharmaceutical companies, donors, and government representatives, met in London and pledged to work together to accelerate the control, prevention, elimination, and eradication of ten NTDs [3] by signing the London Declaration in support of the WHO Roadmap.

In 2015, increased donations of essential medicines have enabled access to treatment for almost a billion people, mainly through preventive chemotherapy for at least one NTD (billion here is defined as a thousand million) [4]. Targeted funding by international developmental agencies and private foundations, domestic financing of control programmes by endemic countries, and increased advocacy and exposure have shifted the world closer to eliminating many of these conditions and improving the lives of millions of “voiceless” people worldwide.

## Progress is evident

In 2015, all Latin American countries achieved universal blood screening for Chagas disease among blood donors. Dracunculiasis is poised for eradication, with a total of only 25 human cases (down from 1,797 human cases in 2010) from only three countries still reporting transmission. Mali reported zero cases for the first time in 2016.

Elimination of human African trypanosomiasis (sleeping sickness) as a public health problem is in sight, with fewer than 3,000 cases reported worldwide in 2015 [5], down from approximately 10,000 in 2009 and nearly 40,000 in 1998. Trachoma has been eliminated as a public health problem in Morocco (2016) and Oman (2012). With more countries moving towards disease elimination, the WHO published new procedures in 2016 on the verification of elimination of onchocerciasis and trachoma [6].

In 2016, the WHO certified six countries—Cambodia, Cook Islands, Maldives, Niue, Sri Lanka, and Vanuatu—as having eliminated lymphatic filariasis as a public health problem [7]. A further 12 countries [8] have now reduced filarial infections to levels that no longer require mass drug administration, and more are expected to achieve elimination targets soon.

In the region of the Americas, onchocerciasis has been almost eliminated; Guatemala (2016), Mexico (2015), Ecuador (2014), and Colombia (2013) have recently been declared free of the disease [9].

While tackling NTDs prioritises the public health needs of poor people, it may be time to revisit the fundamentals of preventive interventions for vector control, veterinary public health, and measures associated with safe water, sanitation, and hygiene.

Clearly, the strategies and responses developed more than a decade ago to control or eliminate NTDs are now woven into the fabric of the 2030 agenda for achieving the Sustainable Development Goals (SDGs). Not only are there many areas of alignment, but many of the same NTD programmes and interventions have implications for multiple goals.

Preventive chemotherapy, for example, has a bearing on poverty, nutrition, education, employment, and gender equality. Combating vector-borne diseases through the provision of safe water or safe water storage has an impact on making cities safer, whereas strengthening linkages between veterinary, environmental, and medical communities can better focus attention on emerging diseases and tackle antimicrobial resistant pathogens [10].

Many of the challenges presented by NTDs require the kind of multisectoral responses encouraged by the 2030 agenda as demonstrated by the numerous alliances working in partnership “to end the epidemic of NTDs.” Nowhere is the issue of integration more pertinent than in relation to goal three of the SDGs—the health goal—which requires the alignment of multiple programmes and initiatives towards the overarching goal of universal health coverage.

The notion of equity woven into the fabric of the NTD agenda reminds the international community that prioritisation of services and explicit consideration must be given to the most disadvantaged groups, including those living in low-income and rural communities who are most at risk of NTDs.

Today, much depends on medicines donated free of charge or subsidised interventions for the very poorest people and for those living in communities that are often beyond the reach of formal health systems. The WHO Roadmap and initiatives such as the London Declaration are game-changers that share the same engagement in mainstreaming NTDs in the global public health agenda as they continue to contribute to expanding access to health.

Extending universal health coverage will require a shift towards greater decentralisation of health services, including outreach beyond fixed health facilities, in order to provide people-centred integrated health services to all.

Meeting the 2030 SDG targets, while “leaving no one behind,” will largely depend on how we prioritise our fight against NTDs.
